# Multiple genetic lineages of anadromous migratory Mekong catfish *Pangasius krempfi* revealed by mtDNA control region and cytochrome *b*

**DOI:** 10.1002/ece3.9845

**Published:** 2023-02-17

**Authors:** Thuy‐Yen Duong, Ngoc‐Tran Thi Nguyen, Dac Dinh Tran, Thanh Hoa Le, Siti Azizah Mohd Nor

**Affiliations:** ^1^ College of Aquaculture and Fisheries Can Tho University Can Tho City Vietnam; ^2^ Immunology Department Institute of Biotechnology (IBT), Vietnam Academy of Science and Technology (VAST) Ho Chi Minh City Vietnam; ^3^ Graduate University of Science and Technology (GUST), Vietnam Academy of Science and Technology (VAST) Ho Chi Minh City Vietnam; ^4^ Institute of Marine Biotechnology Universiti Malaysia Terengganu Terengganu Malaysia

**Keywords:** genetic diversity, Mekong fish, migration, Pangasiidae, population structure

## Abstract

Population genetic structure of migratory fishes can reflect ecological and evolutionary processes. *Pangasius krempfi* is a critically important anadromous catfish in the Mekong River, and its migration pathways and genetic structure have attracted much interest. To investigate, we quantified the genetic diversity of this species using the control region (D‐loop) and Cytochrome *b* (Cyt*b*) of the mitochondrial genome. Fish were sampled (*n* = 91) along the Mekong tributaries from upstream to estuaries and coastal areas in the Mekong Delta and compared to three samples from Pakse (Laos). The D‐loop haplotype (0.941 ± 0.014) and nucleotide diversity (0.0083 ± 0.0005) were high in all populations, but that of Cyt*b* was low (0.331 ± 0.059 and 0.00063 ± 0.00011, respectively). No genetic difference was detected between populations, indicating strong gene flow and confirming a long migration distance for this species. *Pangasius krempfi* was not genetically structured according to geographical populations but was delineated into three haplogroups, suggesting multiple genetic lineages. The presence of haplogroups in each sampling location implies that migration downstream is random but parallel when the fish enter two river tributaries bifurcating from the main Mekong River. Individuals can also migrate along the coast, far from the estuaries, suggesting a longer migration path than previously reported, which is crucial for maintaining diverse genetic origin and migration pathways for *P. krempfi*.

## Graphical abstract

We used the control region and cytochrome *b* to quantify the genetic diversity and to elucidate historical origins and migratory patterns of *Pangasius krempfi*. Fish were sampled along the Mekong tributaries and coastal areas in the Mekong Delta (Vietnam) and referenced to samples in Pakse (Laos). The results indicate moderate levels of genetic diversity, confirm long migration distances, and suggest multiple historical origins of the species inferred from their delineation into three haplogroups.

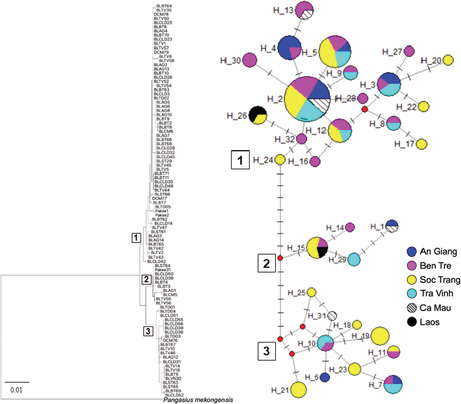

## 1 INTRODUCTION

The Mekong River, one of the world's longest rivers, flowing from the Tibetan plateau in China to Vietnam, has the second‐highest diversity of fish in the world after the Amazon (Durand et al., 2022; Kang & Huang, 2021; Valbo‐Jørgensen et al., 2009). The complicated climatic and geological history of the Mekong River influences the current phylogeographical structure and distribution of its fauna (Sodhi et al., 2004). Among the 1136 Mekong fish species, diadromous fishes (fish that migrate between fresh and marine waters for reproduction) account for only 61 species but are disproportionately economically important (Vu et al., 2020). Diadromous fishes, particularly anadromous species, have wide range of ancestral origins (Bloom & Lovejoy, 2014; Delgado & Ruzzante, 2020; McDowall, 1997) and migration strategies (McDowall, 1997; Vu, Baumgartner, Mallen‐Cooper, et al., 2022). Compared to genetic origin and migration variations between species, within‐species variation has been less documented.

*Pangasius krempfi* Fang & Chaux, 1949, family *Pangasiidae*, is an economically and ecologically important anadromous fish in the Lower Mekong River basin (Baird, 1996; Poulsen et al., 2004). This species has the highest market price compared to other catfish because of its excellent flesh quality (Le & Duong, 2018; Vu et al., 2020), making it the main target for capture fisheries along the Mekong River basin in Laos, Cambodia, and Vietnam (Baird, 1996; Hogan et al., 2007; Roberts & Baird, 1995; Vu et al., 2020). Due to overexploitation, dam building for hydropower, habitat fragmentation, water pollution, and other environmental issues, this species, together with other Mekong fish species, has been dramatically declining (Baran & Myschowoda, 2009; Dugan et al., 2010; Hogan, 2011; Ziv et al., 2012). Consequently, it has been listed as “vulnerable” on the IUCN red list since 2011 (Baird, 2011).

Despite its importance, the ancestral origin and life cycle of this species have not been fully investigated. *P. krempfi* is anadromous, spending its growth stages in the estuaries and coastal areas of the Mekong Delta, Vietnam, with adults migrating to freshwater areas upstream of the Mekong River for spawning (Baird, 1996; Hogan et al., 2007; Poulsen et al., 2004; Roberts & Baird, 1995; Sokheng et al., 1999; Tran et al., 2021; Vu, Baumgartner, Limburg, et al., 2022). However, there is limited information on the downstream migration of this species. Following the upstream migration of *P. krempfi*, spawning is believed to occur from Khone Falls up to the Thailand‐Laos border, at least 720 km from the sea (Hogan et al., 2007; Roberts & Baird, 1995; Sokheng et al., 1999). Recent studies using strontium isotope ratios (^87^Sr/^86^Sr) and the microchemical structure of the otolith found that *P. krempfi* can migrate more than 1400 km upstream (Hogan et al., 2007; Vu, Baumgartner, Limburg, et al., 2022). However, the locations for spawning grounds of this species are still unclear, except for the one near to Khone Falls, which was confirmed by several studies (Baird, 1996; Hogan et al., 2007; Roberts & Baird, 1995; Sokheng et al., 1999; Vu, Baumgartner, Limburg, et al., 2022). Tran et al. (2021) proposed two additional spawning sites in the main stream that have not yet been precisely identified, based on corresponding ^87^Sr/^86^Sr profiles in otoliths of fish collected in the Mekong Delta and in water sampled along the Mekong River. Additionally, the *P. krempfi* spawning season can range from May to early November, as observed in mature individuals at Khone Falls in Laos (Baird, 1996; Sokheng et al., 1999). Such a long spawning season is most likely due to multiple spawning groups returning to the spawning ground at different times. Based on possible spatial and temporal variations in spawning activities of *P. krempfi* (Poulsen et al., 2004; Rainboth, 1996), and its homecoming behavior (Vu, Baumgartner, Limburg, et al., 2022), it is hypothesized that *P. krempfi* originating from different natal spawning sites and/or spawning times could be from different gene pools. Accordingly, genetic divergence among migrating populations would be predicted. We tested this prediction using maternally inherited mitochondrial DNA (mtDNA) markers as described here.

The mitochondrial non‐coding control region (or D‐loop) and protein‐coding gene Cytochrome *b* (Cyt*b*) have been commonly used to study intraspecific genetic diversity in fish (Ochoa et al., 2015; Page & Hughes, 2010; Zhu et al., 1994). In various animal taxa, including fish, the D‐loop generally evolves faster than mtDNA coding genes (Broughton et al., 2001; Brown et al., 1993; Moritz et al., 1987; Page & Hughes, 2010), making it a powerful marker in detecting recent divergence within a species (Grant, 2015). Compared to the D‐loop, Cyt*b* is more conserved, as documented in many fish species, such as the Amazon catfish *Brachyplatystoma platynemum* (Ochoa et al., 2015), feather‐back fish *Chitala chitala* (Mandal et al., 2012), and silver carp *Hypophthalmichthys molitrix* (Chen et al., 2019). The combined use of mtDNA segments with unequal mutation rates reflects a broad view of the evolutionary history of the species (Ballard & Rand, 2005; Moritz et al., 1987; Page & Hughes, 2010). Previous studies on the genetic diversity of pangasiid catfish (family Pangasiidae) have used a single locus, for example, the D‐loop for Mekong giant catfish *Pangasianodon gigas* (Ngamsiri et al., 2007), and Cyt*b* for *Pangasius pangasius* and *Pangasianodon hypophthalmus* (Ha et al., 2020; Mohindra et al., 2015), and 16 S rRNA for nine pangasiid species (Na‐Nakorn et al., 2006). In another study on *P. krempfi*, inter‐simple sequence repeat (ISSR) markers were utilized to test genetic differentiation between populations from two estuaries of the Tien and Hau Rivers (the main Mekong tributaries in the Mekong Delta of Viet Nam). The differentiation between the two populations suggested that they were from separate spawning groups (Duong & Nguyen, 2019). This prediction should be further verified.

In this study, we used two mtDNA loci, D‐loop and Cyt*b*, for estimating the genetic diversity of *P. krempfi*. This approach was used to test a prediction of genetic divergence among migrating populations for this species and provide genetic evidence of its migration pathway, especially downstream, in the Mekong Delta. This information has important implications for the management of this vulnerable species.

## 2 MATERIALS AND METHODS

### 2.1 Ethics statement

All of the fish samples utilized in this investigation were obtained from fishermen or market vendors. The fish were dead at the time of sampling; therefore, no ethical approval was required.

### 2.2 Sampling time and location

**FIGURE 1:**
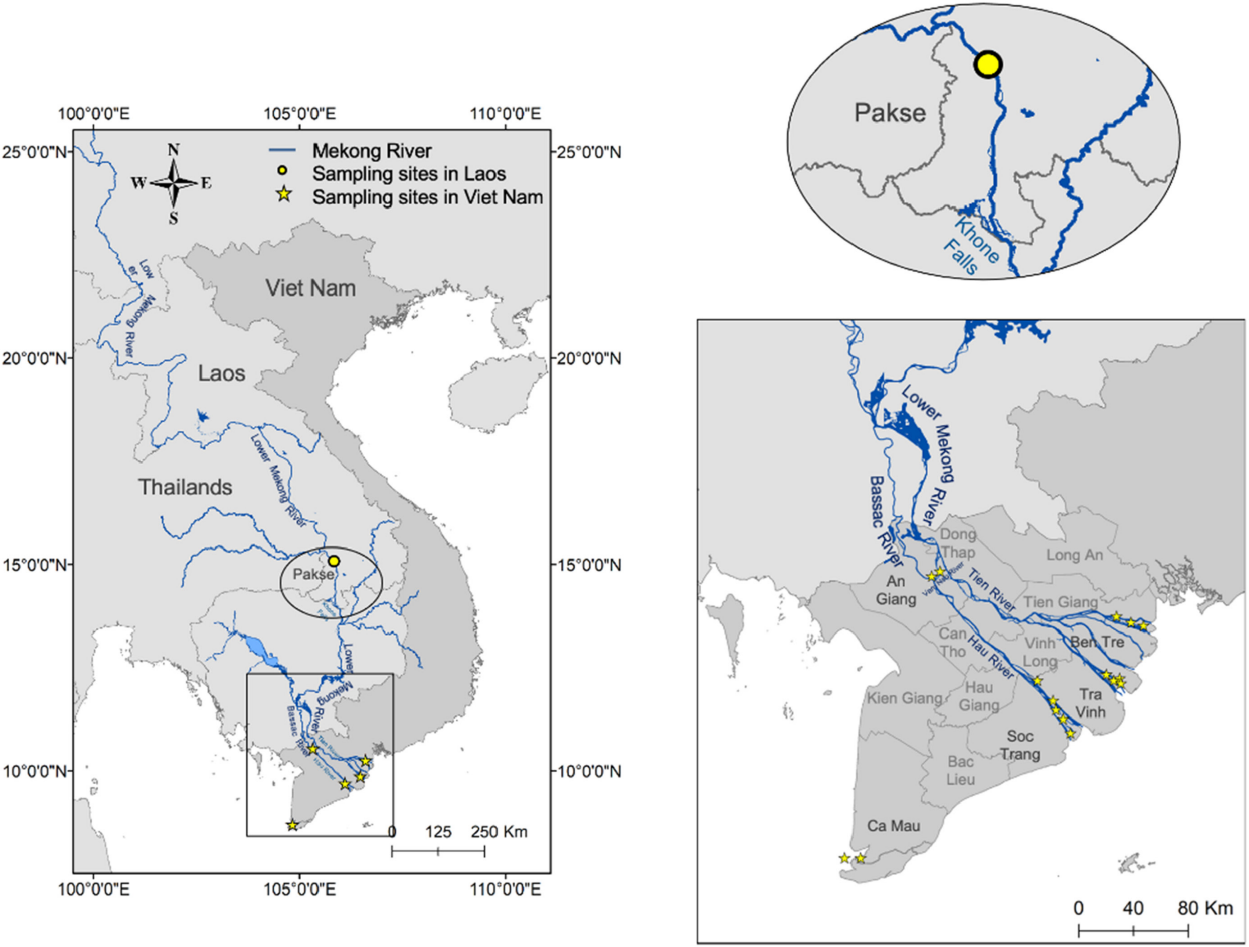
Sampling sites of *Pangasius krempfi* in Vietnam Mekong Delta and Laos.

A total of 91 fish samples (Table 1) were bought from fishermen or in local markets along rivers (where the fish were collected within local areas) in five locations in the Mekong Delta (MD) of Viet Nam (Figure 1). Of these, four locations were along the Mekong River tributaries (Tien and Hau Rivers), including (i) an upstream section located in Vam Nao, An Giang (AG); (ii) Binh Dai, Ben Tre (BT), and (iii) Tra Vinh (TV) in the Tien River; and (iv) Tran De, Soc Trang (ST) in the Hau River. Fish samples were also collected in coastal areas of Ca Mau (CM), representing a non‐Mekong River population. Three samples from Pakse, Laos, in middle Mekong River were also used for comparison. Fish size ranged from 0.0042 to 9.9 kg (Appendix S1: Table S1). Specifically, fish from AG, CM, and Laos had large sizes (1.08–7.05 kg), while BT (0.025–0.095 kg) and TV (0.069–0.714 kg) had only smaller sized fish. ST had a mixture of large (maximum of 9.9 kg) and small‐sized fish (0.0042 kg). A fin clip from each individual was collected and stored in 95% ethanol for genetic analyses.

**TABLE 1:** Genetic diversity indices of *Pangasius krempfi* populations based on D‐loop and Cyt*b* sequences.

Population	*N*	D‐loop			Cyt*b*			Concatenated sequence		
		*H*	*H*_d_	π	*H*	*H*_d_	π	*H*	*H*_d_	π
An Giang (AG)	13	7	0.872 ± 0.067	0.0075 ± 0.0021	3	0.410 ± 0.154	0.00066 ± 0.00027	8	0.897 ± 0.067	0.0046 ± 0.0012
Ben Tre (BT)	20	14	0.953 ± 0.033	0.0072 ± 0.0014	2	0.268 ± 0.113	0.00041 ± 0.00017	14	0.953 ± 0.033	0.0044 ± 0.0009
Soc Trang (ST)	31	15	0.935 ± 0.021	0.0092 ± 0.0005	3	0.428 ± 0.096	0.00070 ± 0.00018	16	0.944 ± 0.019	0.0056 ± 0.0003
Tra Vinh (TV)	21	12	0.943 ± 0.029	0.0079 ± 0.0012	3	0.267 ± 0.120	0.00042 ± 0.00020	13	0.948 ± 0.029	0.0047 ± 0.0007
Ca Mau (CM)	6	5	0.933 ± 0.122	0.0097 ± 0.0030	2	0.533 ± 0.172	0.00081 ± 0.00026	5	0.933 ± 0.122	0.0060 ± 0.0018
Laos	3	2	0.667 ± 0.314	0.0066 ± 0.0031	3	1.000 ± 0.272	0.00203 ± 0.00068	3	1.000 ± 0.272	0.0047 ± 0.0020
**Total**		3	0.941 ± 0.014	0.0083 ± 0.0005	6	0.381 ± 0.059	0.00063 ± 0.00011	37	0.953 ± 0.011	0.0051 ± 0.0003

Abbreviations: *H*, number of haplotypes; *H*_d_, haplotype diversity; *N*, sample size; π, nucleotide diversity.

### 2.3 Genetic analysis

DNA was extracted from the fin clips using Wizard® SV Genomic DNA Purification kit (Promega). Extracted DNA was amplified for mitochondrial Cyt*b* and D‐loop genes. Cytochrome *b* was amplified using a universal primer pair L15803/H16461 (Briolay et al., 1998) with PCR composition and thermal cycle conditions based on Tsigenopoulos and Berrebi (2000). The D‐loop region was amplified using the primer pair of D‐loop‐Thr‐F/D‐loop‐Phe‐R with the thermal cycle conditions described by Cheng et al. (2012). PCR products were purified using Wizard SV Gel and PCR Clean‐Up System (Promega) and then sent for DNA sequencing at Apical Scientific Sdn. Bhd, where Sanger sequencing was conducted using ABI PRISM 3730xl Genetic Analyzer (Applied Biosystems).

### 2.4 Data analysis

Sequences of D‐loop and Cyt*b* were aligned using the ClustalW function in MEGA 7 (Kumar et al., 2016). Ambiguous bases were double‐checked for their sequencing quality in Finch TV version 1.4.0 (Geospiza, Inc.; http://www.geospiza.com), edited, and trimmed before further analyses. The fitting nucleotide substitution models that describe the substitution patterns were tested using the statistical model of maximum likelihood, implemented in MEGA 7. Based on the lowest Bayesian information criterion (BIC) scores (Posada & Buckley, 2004), the best nucleotide substitution model was Tamura 3‐parameter model (Tamura, 1992) for D‐loop and the Kimura‐2 parameter model (Kimura, 1980) for Cyt*b*. These models were used to calculate genetic distances within and between groups and the results were the same. For simplicity, only genetic distances based on the Kimura‐2 parameter (K2P) were reported. Additionally, DnaSP 5.0 (Librado & Rozas, 2009) was used to estimate molecular genetic diversity parameters (i.e., the number of segregating sites [S], the number of haplotypes [*H*], haplotype diversity [*H*_d_] (Nei, 1987), and nucleotide diversity [π]) (Nei, 1987; Nei & Li, 1979) for each population. The phylogenetic relationships among individuals and haplotypes were explored via the phylogenetic trees based on the Tamura 3‐parameter model with 1000 bootstrap replicates (using MEGA7) and a Neighbor‐Joining tree using NETWORK software (Fluxus‐engineering.com). One sample of a sister species *Pangasius mekongensis* Gustiano, Teugels & Pouyaud, 2003 (GenBank accession number for *COI*: KT289880) was used as an outgroup in the phylogenetic trees of *P. krempfi*. Data for D‐loop and Cyt*b* were initially analyzed separately, followed by analysis of the concatenated sequences (two D‐loop and Cyt*b* sequences of each sample were combined using the package “apex” (Jombart et al., 2017) in R (R Core Team, 2020)). Outputs of genetic analyses (i.e., genetic diversity, genetic distances, haplotypes, and phylogenetic trees) based on the three sequence datasets were compared.

Population structure was tested with sequence data and haplotype data. Differentiation between pairwise populations was estimated by *F*_ST_ and tested for their significance with 5000 permutations in Arlequin ver. 3.5 (Excoffier & Lischer, 2010). Arlequin was also employed to partition genetic variation within and between populations. In addition, genetic distances based on the number of nucleotide differences and Tamura 3‐parameter were calculated with 1000 bootstrap replicates using MEGA7. The historical demographic expansion was evaluated using Tajima's *D* (Tajima, 1989) and Fu's *Fs* tests (Fu, 1997) and mismatch distribution analysis (Excoffier, 2004; Rogers & Harpending, 1992), which were conducted in Arlequin 3.5.

## 3 RESULTS

### 3.1 Genetic diversity of *P. krempfi*

A total of 94 samples (including 3 from Laos) of *P. krempfi* yielded 32 D‐loop haplotypes with an overall haplotype diversity of 0.941 ± 0.014, and 6 Cyt*b* haplotypes with haplotype diversity of 0.381 ± 0.059. The D‐loop region (904 bp) had 36 segregating sites, including 5 singleton variable sites and 31 parsimony informative sites. The Cyt*b* segment, with the length of 652 bp, had 5 variable sites (2 singleton and 3 parsimony sites). The D‐loop nucleotide diversity of all samples (0.0083 ± 0.0005) was greater than (13.2‐fold) the Cyt*b*‐based nucleotide diversity (0.00063 ± 0.00011). The three samples in Laos were composed of two D‐loop haplotypes and three Cyt*b* haplotypes (Table 1).

The genetic diversity from different locations showed similar trends for both D‐loop and Cyt*b*. ST and CM had the highest nucleotide diversity, followed by AG, while the BT and TV populations had the lowest nucleotide diversity. The pattern of haplotype diversity across populations was slightly different between D‐loop and Cyt*b* data. The D‐loop haplotype diversity values were high in four populations (excepting AG). However, values based on Cyt*b* were lowest in the BT and TV populations. The results of genetic diversity based on the concatenated sequences were highly correlated with the D‐loop data (Pearson *r* = .98 for Haplotype diversity, and *r* = .76 for nucleotide diversity, Table 1).

### 3.2 Genetic structure of *P. krempfi*

**FIGURE 2:**
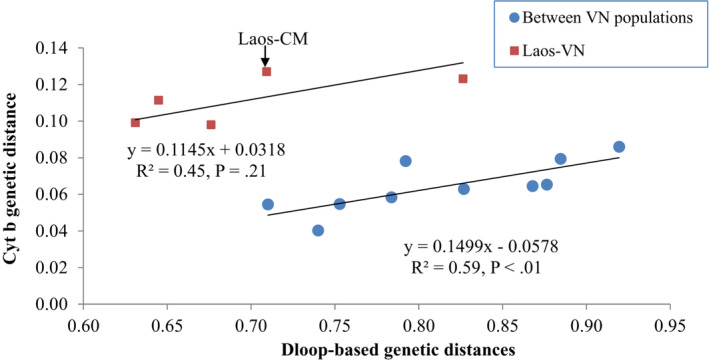
Correlation of K2P genetic distances based on Dloop and Cyt*b* sequences of five populations in Vietnam and one in Laos.

Pairwise K2P genetic distances between populations varied from 0.71% to 0.93% based on D‐loop (Table 2), corresponding to 6.3 and 8.3 nucleotide differences, and from 0.04% to 0.13% based on Cyt*b*, with 1 or 2 nucleotide differences (Table 3). Samples from Laos were most similar to BT, AG, and TV populations based on D‐loop, but more divergent from MD samples based on Cyt*b*. However, both sequences indicated that the ST population was more divergent from the other MD populations, followed by the CM population. When separating out the Laos samples from the other populations, the values of pairwise genetic distances among populations based on the two sequence types were correlated (Figure 2). Genetic differentiation based on both D‐loop and Cyt*b* sequences shows that all pairwise *F*_ST_ values between populations were not statistically different from zero except for the Laos‐BT pair based on D‐loop sequences (Table 4). Even so, the Laos population was more differentiated from the MD populations than pairwise population comparisons within the MD group. The mean *F*_ST_ values were 0.009 (*p* = .117) for D‐loop and higher for Cyt*b* (0.056, *p* = .05). AMOVA analyses show that genetic variation is mainly contributed from within populations, 99% for D‐loop and 94% for Cyt*b* (Table 5).

**TABLE 2:** Kimura‐2 parameter genetic distances (%, mean: below diagonal, SE: above diagonal) between populations based on D‐loop.

Population	AG	BT	ST	TV	CM	LAOS
An Giang (AG)		0.156	0.198	0.163	0.173	0.194
Ben Tre (BT)	0.710		0.195	0.162	0.170	0.192
Soc Trang (ST)	0.902	0.884		0.197	0.201	0.200
Tra Vinh (TV)	0.752	0.739	0.890		0.176	0.191
Ca Mau (CM)	0.791	0.783	0.930	0.825		0.193
Laos	0.797	0.769	0.874	0.802	0.807	

**TABLE 3:** Kimura‐2 parameter genetic distances (%, mean: below diagonal, SE: above diagonal) between populations based on Cyt*b.*

Population	AG	BT	ST	TV	CM	LAOS
An Giang (AG)		0.038	0.041	0.034	0.058	0.078
Ben Tre (BT)	0.055		0.041	0.033	0.057	0.079
Soc Trang (ST)	0.072	0.058		0.037	0.060	0.082
Tra Vinh (TV)	0.055	0.040	0.058		0.055	0.077
Ca Mau (CM)	0.078	0.058	0.080	0.063		0.086
Laos	0.129	0.109	0.131	0.114	0.119	

**TABLE 4:** *F*_ST_ based on Dloop (below diagonal) and Cyt*b* (above diagonal).

Population	AG	BT	ST	TV	CM	LAOS
An Giang (AG)		0.023	0.062	0.016	0.066	0.228
Ben Tre (BT)	−0.045		0.045	−0.03	−0.004	0.382
Soc Trang (ST)	0.038	0.039		0.049	0.052	0.215
Tra Vinh (TV)	−0.023	−0.022	0.015		0.068	0.362
Ca Mau (CM)	−0.092	−0.068	−0.037	−0.064		0.205
Laos	0.168	0.214**	0.189	0.174	0.053	

*Note*: All values were not significantly different from zero (*p* > .05), except a significant value with two asterisks (**) (*p* < .01), based on 1000 permutation tests in Arlequin.

**TABLE 5:** AMOVA results based on D‐loop and cytochrome *b* of *Pangasius krempfi* populations in Vietnam and Laos.

Source of variations	df	Sum of squares	% of variation	Fixation index
Based on D‐loop				
Among populations	5	21.6	0.90	*F*_ST_ = 0.009 *p* = .178
Within populations	82	315.5	99.10	
Based on Cyt*b*				
Among populations	5	1.82	5.62	*F*_ST_ = 0.056 *p* = .050
Within populations	82	16.45	94.38	

### 3.3 Haplotype diversity and structure of *P. krempfi*

**FIGURE 3:**
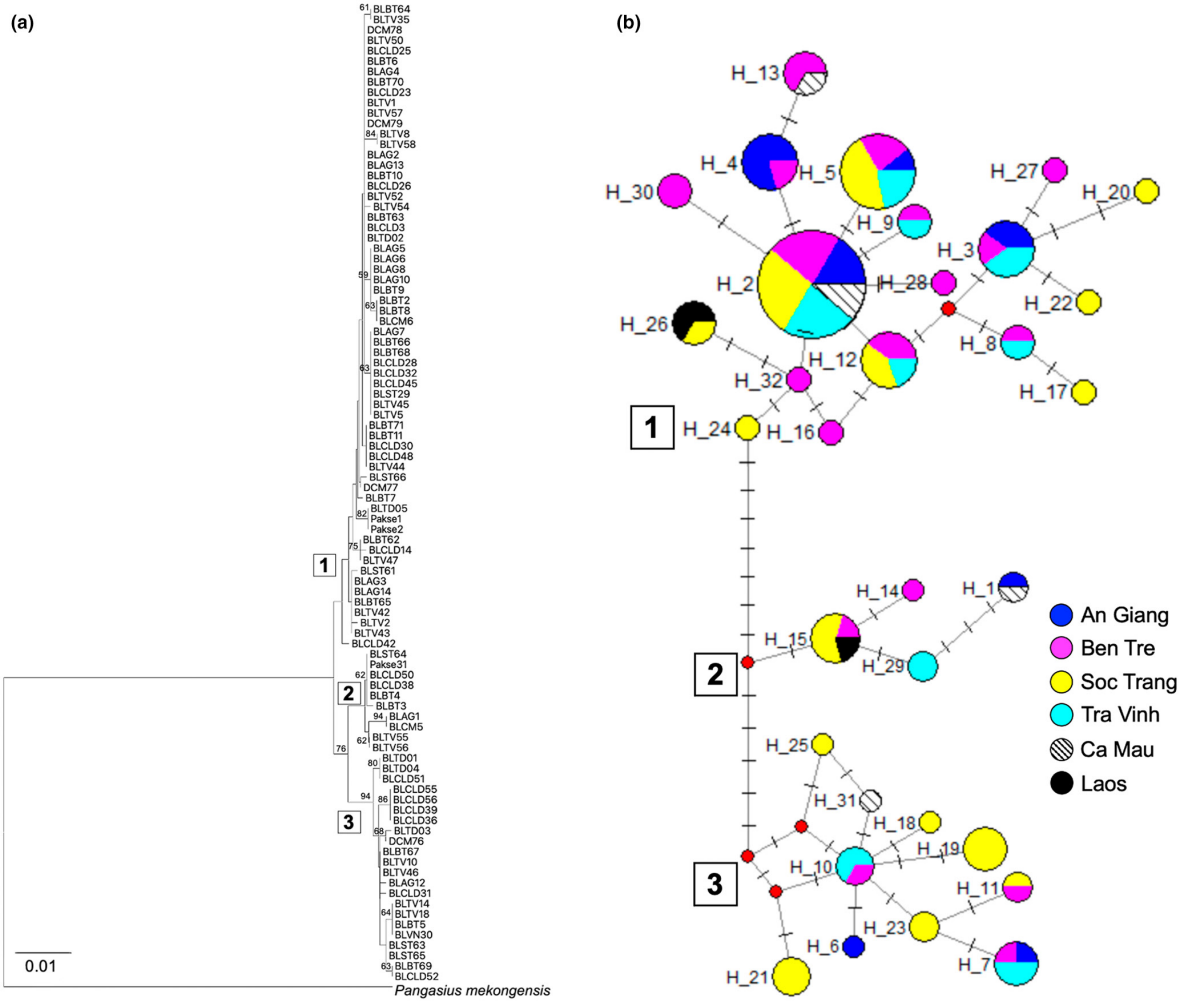
Phylogenetic relationships among D‐loop sequence (*N* = 94) and haplotypes (*N* = 32) of *Pangasius krempfi*. (a) Neighbor‐Joining tree based on D‐loop sequence using the Tamura 3‐parameter method with the bootstrap test (1000 replicates) showing values greater than 50% above the branches. A sister species *Pangasius mekongensis* was used as an outgroup. (b) Median‐joining D‐loop haplotype network of five MD populations and two samples in Laos. The node size is proportional to the number of individuals. Red dots represent median vectors (inferred and unsampled haplotypes). The number in square boxes indicates the haplogroups.

**FIGURE 4:**
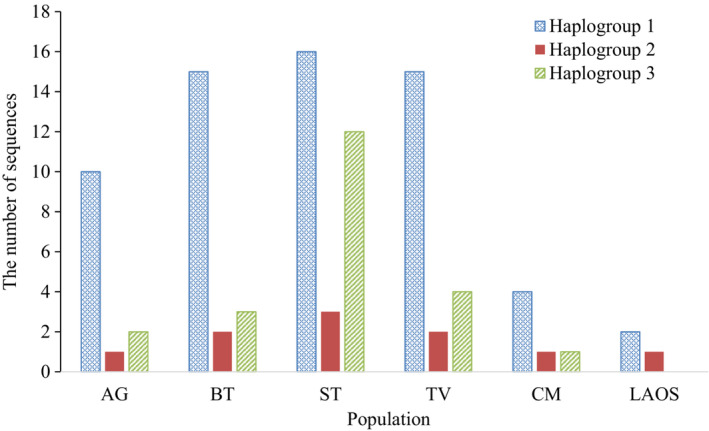
The number of D‐loop sequences from six *Pangasius krempfi* populations in three haplogroups indicated in Figure 3.

Among the 32 D‐loop haplotypes, 14 (43.8%) were shared in at least two populations. Hap‐2 was the most common in the MD populations with a sequence frequency of 19.1%. The ST population had the highest number of unique haplotypes (9/15 D‐loop haplotypes), followed by TV (4/12 D‐loop haplotypes). The lowest numbers were found in AG (1/7 D‐loop haplotypes). Despite the high proportion of population‐specific haplotypes, the phylogenetic relationship of the fish was not structured by population but by three groups of haplotypes (haplogroups) (Figure 3). Haplogroup 1 had the highest number of haplotypes and sequences (18 haplotypes and 62 sequences), followed by Haplogroup 3 (10 haplotypes and 22 sequences), and Haplogroup 2 (4 haplotypes and 10 sequences) (Figure 4). The mean genetic distance between haplogroups (ranged from 1.169% to 1.552%, Table 6) was 1.63‐fold higher than that between populations. The nucleotide differences between haplogroups are from 10.5 to 13.8. Pairwise *F*_ST_ among these three haplotypes groups are high, from 0.707 to 0.768 (mean *F*_ST_: 0.754, *p* < .01).

**TABLE 6:** Genetic distances (mean ± SE) based on Kimura‐2 parameter % (below diagonal) and nucleotide differences (above diagonal) among three D‐loop haplogroups.

Haplogroup	Haplogroup_1	Haplogroup_2	Haplogroup_3
Haplogroup_1		11.47 ± 3.11	13.82 ± 3.44
Haplogroup_2	1.285 ± 0.330 (11.47)		10.45 ± 2.79
Haplogroup_3	1.552 ± 0.369 (13.82)	1.169 ± 0.397 (10.45)	

**FIGURE 5:**
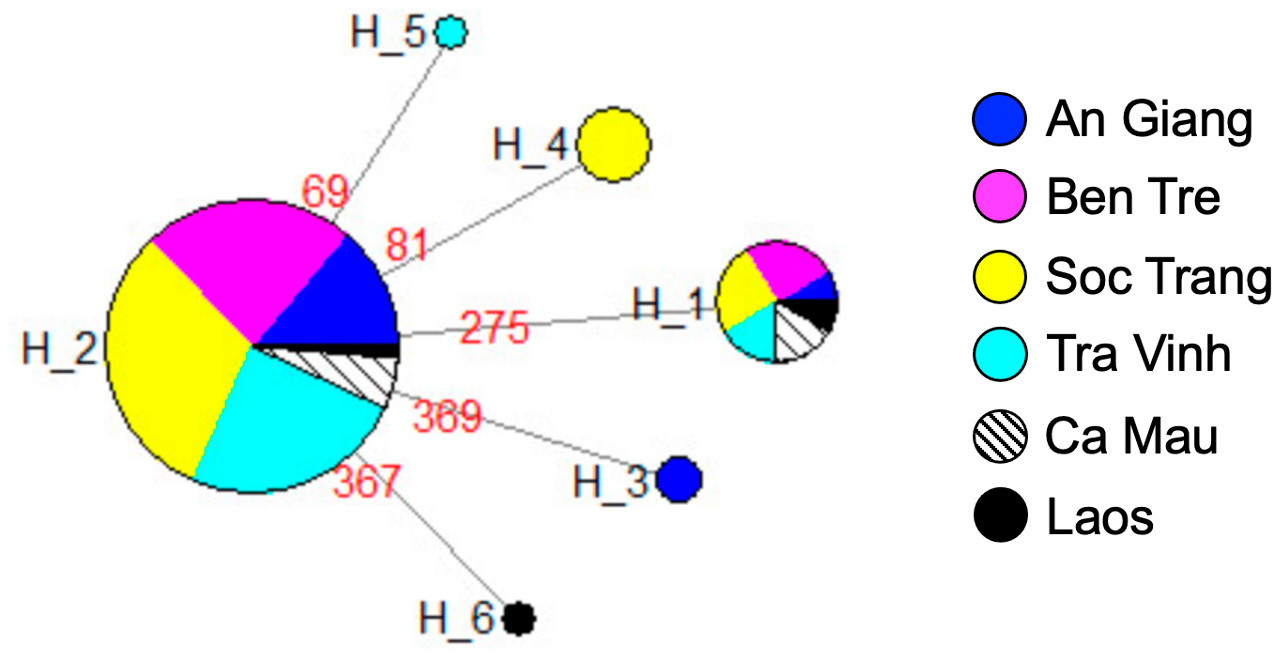
Median‐joining network of six Cyt*b* haplotypes from five MD populations and three samples in Laos. The red numbers indicate mutation sites. Color coding for populations and other notes are similar to those in Figure 3.

Two (out of 6) Cyt*b* haplotypes were shared by all the MD populations (Figure 5), of which *H*_2 was the most common (accounting for 77.7% Cyt*b* sequences, and also observed in Laos). The other five haplotypes differed from the dominant *H*_2 by one nucleotide and from each other by 2 nucleotides.

The concatenated sequences combining D‐loop and Cyt*b* yielded 37 haplotypes, which were also segregated into three haplogroups (data not shown), similar patterns to D‐loop haplotypes network in Figure 3.

### 3.4 Historical demography

Neutrality tests (Table 7) for population demographic expansion showed that, based on the D‐loop sequence, Tajima's *D* values ranged from negative −0.543 (CM) to positive 0.974 (ST), and the range of Fu's *Fs* from −3.553 (BT) to 1.252 (AG). None of these numbers, nor the sum of squared deviation (SSD) or the raggedness index, are significant (*p* > .05), showing that there is no evidence of demographic expansion in either geographic population. The statistical tests based on Cyt*b* showed similar results, except for the TV population with significant negative Fu's *Fs* (*F*s = −1.159, *p* = .036).

**TABLE 7:** Neutrality and goodness‐of‐fit test for *Pangasius krempfi* populations in the Mekong Delta, based on D‐loop and Cyt*b.*

Populations	D‐loop						Cyt*b*					
	Tajima's *D*	*p*‐Value	*F*s	*p*‐Value	SSD	*r*	Tajima's *D*	*p*‐Value	*F*s	*p*‐Value	SSD	*r*
An Giang (AG)	−0.374	0.378	1.252	0.743	0.059	0.070	−0.909	0.220	−0.790	0.087	0.008*	0.171
Ben Tre (BT)	−0.283	0.416	−3.553	0.072	0.034	0.025	−0.861	0.325	0.381	0.363	0.024	0.157
Soc Trang (ST)	0.974	0.874	−0.087	0.521	0.020	0.029	0.046	0.644	0.102	0.407	0.015	0.164
Tra Vinh (TV)	0.253	0.625	−0.762	0.382	0.028	0.032	−1.159	0.184	−1.259	***0.036*** *	0.012	0.163
Ca Mau (CM)	−0.543	0.328	0.762	0.576	0.129	0.200	0.851	0.884	0.625	0.487	0.031	0.289

Abbreviations: *r*, Raggedness index; SSD, sum of squared deviation.

* Indicates significance of *p* < .05 (bold value).

**FIGURE 6:**
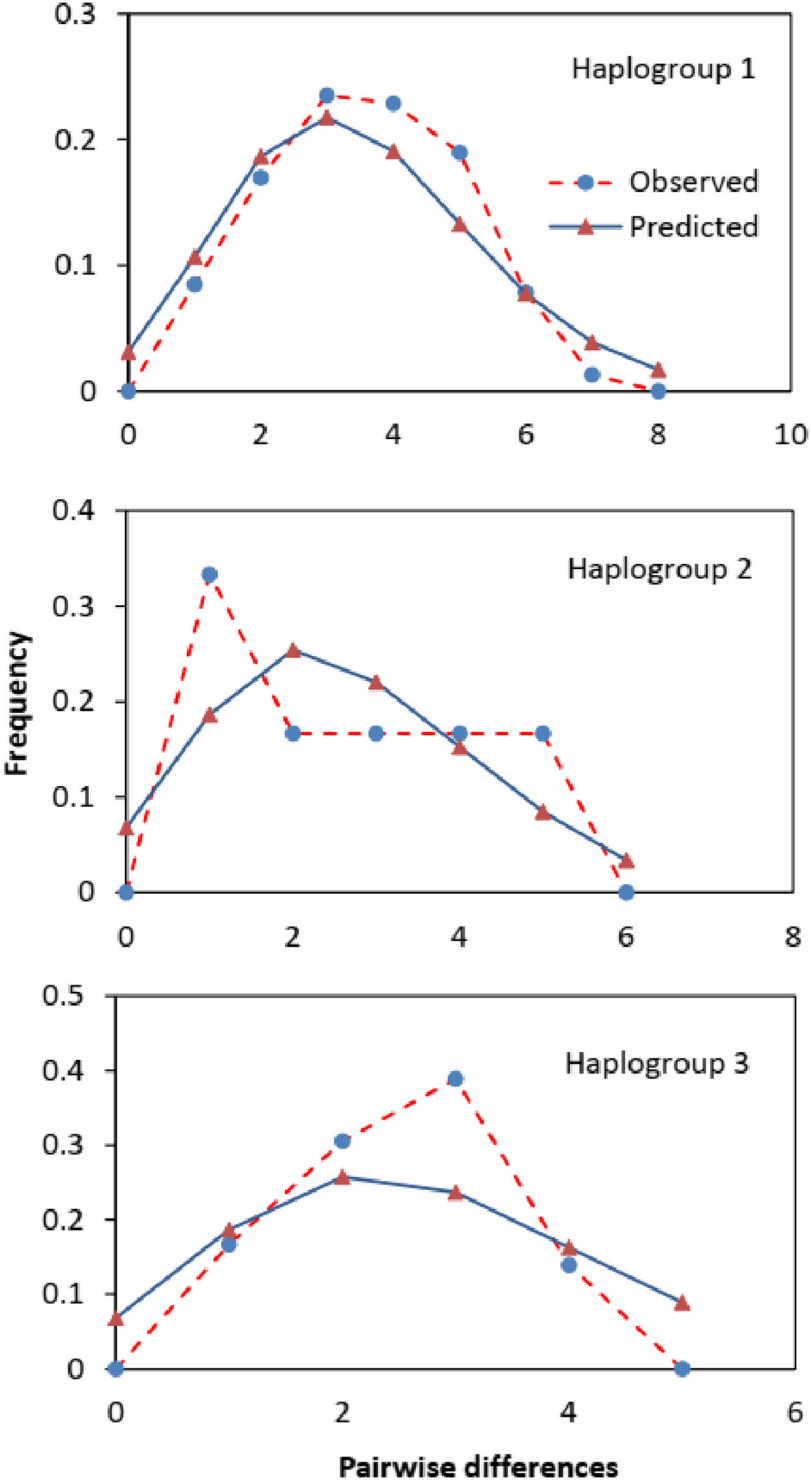
Mismatch distribution analysis of three D‐loop haplogroups of *Pangasius krempfi*.

For the three haplogroups of the D‐loop (Table 8), groups 1 and 3 have negative and significant values of Tajima's *D* (−1.078, *p* = .147 and −1.492, *p* = .049) and Fs (−19.92 and −7.76, *p* < .01). Haplogroups 1 and 3 had a unimodal distribution of pairwise nucleotide differences between individuals and no significant difference between the observed and expected data. Haplogroup 2 had a flattened distribution (Figure 6). Goodness‐of fit tests indicate the observed mismatch distributions were compatible with the simulated (predicted) distributions.

**TABLE 8:** Neutrality and goodness‐of‐fit tests for three D‐loop haplogroups of *Pangasius krempfi*.

Haplogroup	Tajima's *D*	*p*‐Value	Fu's *F*s	*p*‐Value	SSD	*r*
Haplogroup_1	−1.078	0.147	−19.92	<.01**	0.007	0.037
Haplogroup_2	−0.212	0.557	−1.414	.066	0.041	0.167
Haplogroup_3	−1.492	0.059	−7.760	<.01**	0.045	0.136

*Note*: No significance of SSD and *r* were found (*p* > .05).

Abbreviations: *r*, Raggedness index; SSD, sum of squared deviation.

** Indicates significance of *p* < .01 (bold value).

## 4 DISCUSSION

The analyses of mtDNA control region and Cyt*b* indicated relatively high haplotype and nucleotide genetic diversity, and significant genetic differentiation within and among migratory populations of *P. krempfi*. These results revealed important inferences about the historical genetic background and migration patterns of an anadromous species.

### 4.1 Genetic diversity

Based on the categories of genetic diversity suggested by Grant and Bowen (1998), overall genetic diversity of *P. krempfi* (excluded three samples from Laos) was low in both haplotype (*H*_d_ < 0.50) and nucleotide diversity (π < 0.005) for Cyt*b*, high in both for the D‐loop (*H*_d_ from 0.872 to 0.953; pi from 0.0072 to 0.0098), with high *H*_d_ and moderate π for the concatenated sequences (Table 1). The level of genetic diversity for *P. krempfi* was evaluated by comparing it with other species using the same DNA segments (Grant & Bowen, 1998). In an endangered Mekong catfish species *Pangasianodon gigas* (the same family Pangasiidae), two populations in Thailand and Cambodia had D‐loop haplotype diversity (*H*_d_: 0.89 and 0.79) and nucleotide diversity (π: 0.0087 and 0.0075, respectively) (Ngamsiri et al., 2007) comparable to those of *P. krempfi* in the current study (D‐loop *H*_d_ = 0.939 and π = 0.0081). Two migratory catfish, *Pseudoplatystoma corruscans* and *Pseudoplatystoma reticulatum*, also had similar levels of D‐loop *H*_d_ (0.814 and 0.952, respectively) and π (0.007) to those of *P. krempfi*; however, *Pseudoplatystoma* spp. populations were found to be more abundant. Other catfish species had lower genetic diversity than *P. krempfi*, such as the threatened freshwater catfish *Tandanus tandanus* in Australia with a D‐loop *H*_d_ = 0.672 and π = 0.006 (Hill et al., 2015), and *Clarias macrocephalus* in the Philippines with D‐loop *H*_d_ = 0.479 and π = 0.00058 (Tan et al., 2016) and in Malaysia with concatenated D‐loop‐Cyt*b H*_d_ from 0.657 to 0.765 and π = 0.003 (Nazia et al., 2010). The above comparisons indicate that *P. krempfi* still harbors moderate‐to‐high genetic diversity in the populations investigated comparable to other rare or abundant fish species. However, high levels of mtDNA genetic diversity do not necessarily indicate large population size (Bazin et al., 2006). *P. krempfi* has been classified “vulnerable” since 2011 (Baird, 2011) and has recently encountered increased challenges, mostly as a result of dam barriers impeding migration routes, overexploitation, and habitat degradation (Nuon et al., 2020; Ziv et al., 2012). A survey in 2017 along estuary areas in the Mekong Delta reported that interviewed fishermen observed a decline in catch and smaller harvest sizes of *P. krempfi* (Le & Duong, 2018). Therefore, management efforts should be prioritized for this vulnerable species.

### 4.2 Genetic structure of *P. krempfi* reveals its migration and historical origin

All populations of *P. krempfi* in the Mekong Delta shared common haplotypes for the D‐loop and Cyt*b*, and had low, nonsignificant genetic difference parameters, including K2P genetic distances and *F*_ST_, indicating that gene flow is high among these populations. These results align with the migratory behavior of the species (Hogan et al., 2007; Poulsen et al., 2004). Additionally, the sharing of D‐loop haplotypes and nonsignificant genetic differences (*F*_ST_) between Laos and Vietnamese populations (D‐loop and Cyt*b*) suggests a long migration distance for the species with a projected longest path (based on samples collected in the present study) from Paske (Laos) to Ca Mau (Vietnam) of approximately 1070 km. This genetic evidence is consistent with findings based on isotope analysis and microchemical signals of the otolith, which showed that *P. krempfi* can migrate at least 720 km (Hogan et al., 2007), or even farther, beyond 1400 km (Tran et al., 2021; Vu, Baumgartner, Limburg, et al., 2022). Previous studies (Hogan et al., 2007; Tran et al., 2021) estimated migration distances from the upstream Mekong River in Laos to estuaries. In the present study, we found no significant genetic difference between CM fish, far from the estuaries, and those from the Mekong River and Laos (Table 4 and Figure 2), suggesting a longer migration path than previously reported. Like *P. krempfi*, other pangasiid species have long migratory behaviors, resulting in genetic homogeneity among populations, such as in *Pangasius bocourti*, *Pangasianodon hypophthalmus* (So et al., 2006), and *Pangasianodon gigas* (Ngamsiri et al., 2007).

Genetic differences between Vietnamese populations of *P. krempfi* reflect migration pathways of the species in the Lower Mekong River. When fish migrate from the Mekong River downstream to Phnom Penh (Cambodia), about 330 kilometers from the sea, where the river splits into two (Pantulu, 1986; Van Zalinge et al., 2003), they can enter either the Bassac River (which flows in the same direction as the Hau River in Vietnam) or the Mekong River (the same flow as the Tien River). Even though the two rivers are linked by the Vam Nao River (An Giang), downstream migration in the two rivers can be parallel, which is supported by the finding that the ST population in the Hau River was less genetically related (based on both D‐loop and Cyt*b*) to the Tien River's other two populations (BT and TV). This result is consistent with a previous study using ISSR markers, which found a low genetic structure between the two fish groups in Tien and Hau estuaries (Duong & Nguyen, 2019). Moreover, each sampling location contained all three haplogroups, implying that migration downstream is random as the fish enters the two river tributaries bifurcating from the Mekong River.

Interestingly, the phylogenetic trees (Neighbor‐Joining tree and Median‐joining haplotype network) constructed based on the D‐loop show that *P. krempfi* was not genetically structured by geographic populations but by three haplogroups (Figure 4). Concatenated sequences yielded the same results (data not shown). Haplogroup 2 had only one haplotype (*H*_1) of Cyt*b*, while haplogroups 1 and 3 shared the same haplotype (*H*_2) of Cyt*b*. The three haplogroups are distinct and differ from the others with high *F*_ST_ values (0.707 to 0.768, *p* < .01). In addition, genetic distance between haplogroups was much higher (1.63‐fold) than that between sampling populations. These results suggest multiple genetic lineages of the species. Because of the small sample sizes from some locations, it is possible that the three haplogroups would become less differentiated as more individuals were added. However, the three distinct D‐loop haplogroups were observed in each of all downstream sampling populations (Figure 4), in each weight class across sampling years (Appendix S2: Figure S1), each year of all size classes (Appendix S2: Figure S2) or in size classes adjusted by sampling years (Appendix S2: Figure S3). These analyses indicate that the finding of multiple genetic lineages of *P. krempfi* populations would be robust. Previous studies based on morphological observation hypothesized that *P. krempfi* distributed along the Mekong River basin could include two distinct populations or species (Poulsen et al., 2004; Rainboth, 1996). Based on three distinct strontium isotopes in the otoliths, Tran et al. (2021) suggested that *P. krempfi* has three natal origins in freshwater areas along the Mekong River. Although the natal spawning sites mentioned in that study should be further verified, the main conclusion on multiple origins agrees with our findings and those from another ISSR markers study (Duong & Nguyen, 2019).

Each haplogroup consisted of representative samples from all populations except the Laos population (likely due to a small sample size). However, haplogroups 1 and 3 had higher numbers of haplotypes (Figure 4) and sequences than those of haplogroup 2 (Figure 6). In addition, they had significant negative values of Tajima's *D* and Fs (Table 8), indicating that these two haplogroups have previously undergone population expansion. Na‐Nakorn et al. (2006) reported that nine pangasiid species in their study were also under population expansion, inferred from the mitochondrial 16S rRNA gene. The expansion period for pangasiid catfish species was estimated from 0.08 to 1.44 Mya (Na‐Nakorn et al., 2006), while many other fishes were from 0.02 to 0.20 Mya, before or during the last glacial maximum (Grant, 2015). The existence of multiple haplogroups could be a result from the history of Mekong which was previously connected with other river systems or fragmented into few different paleo rivers (Voris, 2000; Woodruff, 2010; Zhang et al., 2019). Other freshwater fishes in the Southeast Asian region such as chevron snakehead fish *Channa striata* and tire track eel *Mastacembelus favus* were also reported to comprise of different lineages, which were explained by the geographic changes during Pleistocene (Adamson et al., 2012; Jamaluddin et al., 2019). Thus, all of the genetic patterns observed in previous and current studies can be attributed to the Mekong River's history of fragmentation and amalgamation (Jamaluddin et al., 2019; Takagi et al., 2011).

### 4.3 Comparing D‐loop and Cyt*b*

In many fish taxa, the D‐loop has higher mutation rates compared to other mtDNA genes such as Cyt*b* (Broughton et al., 2001; Brown et al., 1993; Mcmillan & Palumbi, 1997; Zhu et al., 1994). This is also true for *P. krempfi* as its D‐loop has a 13.2‐fold greater nucleotide diversity than Cyt*b* (Table 1). Due to its conserved nature, genetic differentiation (*F*_ST_) based on Cyt*b* across populations (Table 4) was larger than that based on the D‐loop. Similarly, higher within‐population nucleotide diversity and smaller between‐population genetic differences were inferred from D‐loop compared to Cyt*b*. This has also been reported for other freshwater fish species such as the feather‐back fish *Chitala chitala* (Mandal et al., 2012), snakehead *Channa striata* (Duong et al., 2019), silver carp *Hypophthalmichthys molitrix* (Chen et al., 2019). Although the two sequence types differ in evolution rates, we found that, in general, they both yielded positive relationships in nucleotide diversity (Table 1), K2P genetic distances (Figure 2), and *F*_ST_ (Table 4). However, the positive correlation in pairwise K2P genetic distance between D‐loop and Cyt*b* is different between two comparison groups, in which pairwise testing showed that Laos‐MD populations had larger genetic distances compared to populations within the MD.

To date, few studies have focused on comparing genetic distance estimates from D‐loop and Cyt*b* within a fish species. Some studies analyzed concatenated segments of the two sequences (Ju et al., 2021; Nazia et al., 2010) but did not mention the results for a single locus. At an interspecific level, Zhu et al. (1994) found that the sequence divergence among sister species of genus *Melanotaenia* (rainbowfish in Australia), estimated from D‐loop and Cyt*b*, was positively correlated. Page and Hughes (2010) compared the performance of the D‐loop and three coding genes *COI*, *ATP*, and Cyt*b* in K2P genetic distance analysis of Australian freshwater fish. The authors reported that the correlations of the D‐loop with K2P distance matrices of *COI*, *ATP*, and Cyt*b* were positive but not significant (*p* > .05), except for D‐loop vs. ATP in genera *Melanotaenia* (rainbowfish, family Melanotaeniidae) and *Retropinna* (cucumberfish, family Retropinnidae). Like the above studies, positive relationships between D‐loop and Cyt*b* were found in estimates for all genetic parameters of *P. krempfi*. These concordant outputs from D‐loop and Cyt*b* analyses strengthen our findings.

In conclusion, *P. krempfi* has moderate‐to‐high levels of genetic diversity. Low genetic differences and haplotype sharing across populations confirm a long migration pathway from the upstream Mekong River in Laos to estuaries and coastal areas in the Mekong Delta. Three high‐divergence haplogroups suggest multiple genetic lineages for this species with variable historical demography.

The findings from the current study can be tested in a further work with more samples and more upstream sampling locations along the Mekong River, especially between Vietnam and Laos. In addition, mtDNA sequence variation alone may not represent the precise or comprehensive evolutionary history of *P. krempfi*. Biasness in mtDNA evolution could also occur. Thus, it would be prudent to include other unlinked genetic markers, such as microsatellite, single‐nucleotide polymorphism (SNP), and restriction site‐associated DNA (RAD) sequencing in future studies.

## AUTHOR CONTRIBUTIONS

**Thuy‐Yen Duong:** Conceptualization (equal); data curation (equal); formal analysis (equal); funding acquisition (equal); investigation (equal); methodology (equal); project administration (equal); writing – original draft (equal); writing – review and editing (equal). **Ngoc‐Tran Thi Nguyen:** Data curation (equal); formal analysis (equal); investigation (equal); writing – original draft (equal); writing – review and editing (equal). **Dac Dinh Tran:** Conceptualization (equal); investigation (equal); methodology (equal); writing – review and editing (equal). **Thanh Hoa Le:** Conceptualization (equal); methodology (equal); writing – review and editing (equal). **Siti Azizah Mohd Nor:** Conceptualization (equal); methodology (equal); writing – original draft (equal); writing – review and editing (equal).

## CONFLICT OF INTEREST STATEMENT

The authors declare no conflicts of interest.

## Supporting information


Appendix S1–S2.

## Data Availability

DNA sequences: Genbank accessions ON237747–ON237834 and OQ366553–OQ3665538 for D‐loop, and ON237835–ON237922 and OQ366559–OQ366564 for Cytochrome *b*.
