# Supplementary material for: Multiple genetic lineages of anadromous migratory Mekong catfish Pangasius krempfi revealed by mtDNA control region and cytochrome b
Source: Ecol Evol. 2023 Feb 17;13(2):e9845. doi: 10.1002/ece3.9845 (PMC9937891; doi:10.1002/ece3.9845)
Supplement: Supplementary file 1 — Appendix S1–S2. [file ECE3-13-e9845-s001.docx]

**Supporting information**

**Multiple genetic lineages of anadromous migratory Mekong catfish *Pangasius krempfi* revealed by mtDNA control region and *Cytochrome b***

Thuy-Yen Duong^*^, Nguyen Thi Ngoc Tran, Tran Dac Dinh, Le Thanh, Mohd Nor Siti Azizah

**Appendix S1** Details of sampling information in this study

**Table S1** Sampling information of *Pangasius krempfi*

| **Sample ID** | **Sampling locations** | **Sampling time** | **Weight at catch (g)** | **Groups of weight at catch** | **Size-Adjusted (g)** | **Groups by size-adjusted (by year at catch)** | **D-loop Haplotype** | **D-loop Haplogroup** | **D-loop**  **(GB #)** | ***Cytb* Haplotype** | ***Cytb***  **(GB #)** | **Concatenated Hapgroup** |
| --- | --- | --- | --- | --- | --- | --- | --- | --- | --- | --- | --- | --- |
| BLAG1 | Vam Nao, AG | 01/04/2019 | 3,100 | D | 3,100 | B’ | 1 | II | ON237747 | 1 | ON237835 | 1 |
| BLAG2 | Vam Nao, AG | 01/04/2019 | 7,050 | D | 7,050 | C’ | 2 | I | ON237748 | 2 | ON237836 | 2 |
| BLAG3 | Vam Nao, AG | 01/04/2019 | 4,250 | D | 4,250 | B’ | 3 | I | ON237749 | 2 | ON237837 | 3 |
| BLAG4 | Vam Nao, AG | 01/04/2019 | 2,800 | C | 2,800 | B’ | 2 | I | ON237750 | 3 | ON237838 | 4 |
| BLAG5 | Vam Nao, AG | 01/04/2019 | 4,150 | D | 4,150 | B’ | 4 | I | ON237751 | 2 | ON237839 | 5 |
| BLAG6 | Vam Nao, AG | 16/04/2019 | 2,200 | C | 2,200 | A’ | 4 | I | ON237752 | 2 | ON237840 | 5 |
| BLAG7 | Vam Nao, AG | 16/04/2019 | 2,100 | C | 2,100 | A’ | 5 | I | ON237753 | 2 | ON237841 | 6 |
| BLAG8 | Vam Nao, AG | 16/04/2019 | 2,400 | C | 2,400 | A’ | 4 | I | ON237754 | 2 | ON237842 | 5 |
| BLAG10 | Vam Nao, AG | 16/04/2019 | 3,100 | D | 3,100 | B’ | 4 | I | ON237755 | 2 | ON237843 | 5 |
| BLAG12 | Vam Nao, AG | 16/04/2019 | 2,050 | C | 2,050 | A’ | 6 | III | ON237756 | 2 | ON237844 | 7 |
| BLAG13 | Vam Nao, AG | 16/04/2019 | 2,200 | C | 2,200 | A’ | 2 | I | ON237757 | 3 | ON237845 | 4 |
| BLAG14 | Vam Nao, AG | 16/04/2019 | 2,050 | C | 2,050 | A’ | 3 | I | ON237758 | 2 | ON237846 | 3 |
| BLVN30 | Vam Nao, AG | 16/02/2017 | 5,700 | D | 7,700 | C’ | 7 | III | ON237759 | 2 | ON237847 | 8 |
| BLBT2 | Binh Dai, BT | 26/04/2017 | 89 | A | 2,089 | A’ | 13 | I | ON237770 | 2 | ON237858 | 14 |
| BLBT3 | Binh Dai, BT | 26/04/2017 | 80 | A | 2,080 | A’ | 14 | II | ON237771 | 1 | ON237859 | 15 |
| BLBT4 | Binh Dai, BT | 26/04/2017 | 82 | A | 2,082 | A’ | 15 | II | ON237772 | 1 | ON237860 | 16 |
| BLBT5 | Binh Dai, BT | 26/04/2017 | 85 | A | 2,085 | A’ | 7 | III | ON237773 | 1 | ON237861 | 17 |
| BLBT6 | Binh Dai, BT | 26/04/2017 | 83 | A | 2,083 | A’ | 2 | I | ON237774 | 2 | ON237862 | 2 |
| BLBT7 | Binh Dai, BT | 26/04/2017 | 81 | A | 2,081 | A’ | 16 | I | ON237775 | 2 | ON237863 | 18 |
| BLBT8 | Binh Dai, BT | 26/04/2017 | 86 | A | 2,086 | A’ | 13 | I | ON237776 | 2 | ON237864 | 14 |
| BLBT9 | Binh Dai, BT | 26/04/2017 | 81 | A | 2,081 | A’ | 4 | I | ON237777 | 2 | ON237865 | 5 |
| BLBT10 | Binh Dai, BT | 26/04/2017 | 85 | A | 2,085 | A’ | 2 | I | ON237778 | 2 | ON237866 | 2 |
| BLBT11 | Binh Dai, BT | 26/04/2017 | 82 | A | 2,082 | A’ | 12 | I | ON237779 | 2 | ON237867 | 13 |
| BLBT62 | Binh Dai, BT | 26/04/2017 | 67.91 | A | 2,068 | A’ | 8 | I | ON237760 | 2 | ON237848 | 9 |
| BLBT63 | Binh Dai, BT | 26/04/2017 | 82.32 | A | 2,082 | A’ | 2 | I | ON237761 | 2 | ON237849 | 2 |
| BLBT64 | Binh Dai, BT | 26/04/2017 | 51.35 | A | 2,051 | A’ | 9 | I | ON237762 | 2 | ON237850 | 10 |
| BLBT65 | Binh Dai, BT | 26/04/2017 | 50.96 | A | 2,051 | A’ | 3 | I | ON237763 | 2 | ON237851 | 3 |
| BLBT66 | Binh Dai, BT | 26/04/2017 | 47.37 | A | 2,047 | A’ | 5 | I | ON237764 | 2 | ON237852 | 6 |
| BLBT67 | Binh Dai, BT | 26/04/2017 | 32.79 | A | 2,033 | A’ | 10 | III | ON237765 | 2 | ON237853 | 11 |
| BLBT68 | Binh Dai, BT | 26/04/2017 | 81.94 | A | 2,082 | A’ | 5 | I | ON237766 | 2 | ON237854 | 6 |
| BLBT69 | Binh Dai, BT | 26/04/2017 | 70.17 | A | 2,070 | A’ | 11 | III | ON237767 | 2 | ON237855 | 12 |
| BLBT70 | Binh Dai, BT | 26/04/2017 | 25.11 | A | 2,025 | A’ | 2 | I | ON237768 | 2 | ON237856 | 2 |
| BLBT71 | Binh Dai, BT | 26/04/2017 | 94.9 | A | 2,095 | A’ | 12 | I | ON237769 | 2 | ON237857 | 13 |
| BLCM5 | CM | 09/04/2019 | 2,050 | C | 2,050 | A’ | 1 | II | ON237827 | 1 | ON237915 | 1 |
| BLCM6 | CM | 09/04/2019 | 2,200 | C | 2,200 | A’ | 13 | I | ON237828 | 2 | ON237916 | 14 |
| BLCM76 | CM | 31/07/2014 | 1,100 | C | 4,100 | B’ | 31 | III | ON237829 | 2 | ON237917 | 35 |
| BLCM77 | CM | 31/07/2014 | 1,082 | C | 4,082 | B’ | 32 | I | ON237830 | 1 | ON237918 | 36 |
| BLCM78 | CM | 31/07/2014 | 1,156 | C | 4,156 | B’ | 2 | I | ON237831 | 2 | ON237919 | 2 |
| BLCM79 | CM | 31/07/2014 | 1,415 | C | 4,415 | B’ | 2 | I | ON237832 | 2 | ON237920 | 2 |
| BLCLD3 | Cu Lao Dung, ST | 23/12/2016 | 10.2 | A | 3,010 | B’ | 2 | I | ON237780 | 2 | ON237868 | 2 |
| BLCLD14 | Cu Lao Dung, ST | 23/12/2016 | 4.2 | A | 3,004 | B’ | 17 | I | ON237781 | 2 | ON237869 | 19 |
| BLCLD23 | Cu Lao Dung, ST | 23/12/2016 | 4.4 | A | 3,004 | B’ | 2 | I | ON237782 | 4 | ON237870 | 20 |
| BLCLD25 | Cu Lao Dung, ST | 23/12/2016 | 9.22 | A | 3,009 | B’ | 2 | I | ON237783 | 2 | ON237871 | 2 |
| BLCLD26 | Cu Lao Dung, ST | 23/12/2016 | 5.65 | A | 3,006 | B’ | 2 | I | ON237784 | 2 | ON237872 | 2 |
| BLCLD28 | Cu Lao Dung, ST | 23/12/2016 | 8.82 | A | 3,009 | B’ | 5 | I | ON237785 | 2 | ON237873 | 6 |
| BLCLD30 | Cu Lao Dung, ST | 16/02/2017 | 49.76 | A | 2,050 | A’ | 12 | I | ON237786 | 2 | ON237874 | 13 |
| BLCLD31 | Cu Lao Dung, ST | 16/02/2017 | 38.77 | A | 2,039 | A’ | 18 | III | ON237787 | 2 | ON237875 | 21 |
| BLCLD32 | Cu Lao Dung, ST | 16/02/2017 | 28.51 | A | 2,029 | A’ | 5 | I | ON237788 | 2 | ON237876 | 6 |
| BLCLD36 | Cu Lao Dung, ST | 16/02/2017 | 27.48 | A | 2,027 | A’ | 19 | III | ON237789 | 4 | ON237877 | 22 |
| BLCLD38 | Cu Lao Dung, ST | 16/02/2017 | 26.09 | A | 2,026 | A’ | 15 | II | ON237790 | 1 | ON237878 | 16 |
| BLCLD39 | Cu Lao Dung, ST | 16/02/2017 | 16.36 | A | 2,016 | A’ | 19 | III | ON237791 | 4 | ON237879 | 22 |
| BLCLD42 | Cu Lao Dung, ST | 16/02/2017 | 17.85 | A | 2,018 | A’ | 20 | I | ON237792 | 2 | ON237880 | 23 |
| BLCLD45 | Cu Lao Dung, ST | 16/02/2017 | 26.45 | A | 2,026 | A’ | 5 | I | ON237793 | 2 | ON237881 | 6 |
| BLCLD48 | Cu Lao Dung, ST | 16/02/2017 | 16.96 | A | 2,017 | A’ | 12 | I | ON237794 | 2 | ON237882 | 13 |
| BLCLD50 | Cu Lao Dung, ST | 16/02/2017 | 39.4 | A | 2,039 | A’ | 15 | II | ON237795 | 1 | ON237883 | 16 |
| BLCLD51 | Cu Lao Dung, ST | 16/02/2017 | 21.52 | A | 2,022 | A’ | 21 | III | ON237796 | 2 | ON237884 | 24 |
| BLCLD52 | Cu Lao Dung, ST | 16/02/2017 | 27.69 | A | 2,028 | A’ | 11 | III | ON237797 | 2 | ON237885 | 12 |
| BLCLD55 | Cu Lao Dung, ST | 16/02/2017 | 17.78 | A | 2,018 | A’ | 19 | III | ON237798 | 4 | ON237886 | 22 |
| BLCLD56 | Cu Lao Dung, ST | 16/02/2017 | 17.1 | A | 2,017 | A’ | 19 | III | ON237799 | 4 | ON237887 | 22 |
| BLST29 | Phong Nam, ST | 16/02/2017 | 5,400 | D | 7,400 | C’ | 5 | I | ON237800 | 2 | ON237888 | 6 |
| BLST61 | Phong Nam, ST | 01/08/2018 | 5,900 | D | 5,900 | C’ | 22 | I | ON237801 | 2 | ON237889 | 25 |
| BLST63 | Phong Nam, ST | 20/3/2019 | 3,700 | D | 3,700 | B’ | 23 | III | ON237802 | 2 | ON237890 | 26 |
| BLST64 | Phong Nam, ST | 20/3/2019 | 5,800 | D | 5,800 | C’ | 15 | II | ON237803 | 1 | ON237891 | 16 |
| BLST65 | Phong Nam, ST | 21/03/2019 | 9,900 | D | 9,900 | C’ | 23 | III | ON237804 | 2 | ON237892 | 26 |
| BLST66 | Phong Nam, ST | 21/03/2019 | 4,100 | D | 4,100 | B’ | 24 | I | ON237805 | 2 | ON237893 | 27 |
| BLTD01 | Tran De, ST | 12/05/2022 | 4,500 | D | 469 | A’ | 21 | III |  | 2 |  | 24 |
| BLTD02 | Tran De, ST | 19/06/2022 | 4,900 | D | 680 | A’ | 2 | I |  | 2 |  | 2 |
| BLTD03 | Tran De, ST | 21/06/2022 | 4,300 | D | 520 | A’ | 25 | III |  | 2 |  | 28 |
| BLTD04 | Tran De, ST | 02/07/2022 | 4,700 | D | 700 | A’ | 21 | III |  | 2 |  | 24 |
| BLTD05 | Tran De, ST | 08/08/2022 | 4,600 | D | 610 | A’ | 26 | I |  | 2 |  | 29 |
| BLTV1 | TV | 22/05/2014 | 365 | B | 3,365 | B’ | 2 | I | ON237806 | 2 | ON237894 | 2 |
| BLTV2 | TV | 22/05/2014 | 450 | B | 3,450 | B’ | 27 | I | ON237810 | 2 | ON237898 | 30 |
| BLTV5 | TV | 16/08/2014 | 475 | B | 3,475 | B’ | 5 | I | ON237818 | 2 | ON237906 | 6 |
| BLTV8 | TV | 22/05/2014 | 69.1 | A | 3,069 | B’ | 30 | I | ON237825 | 2 | ON237913 | 34 |
| BLTV10 | TV | 22/05/2014 | 425 | B | 3,425 | B’ | 10 | III | ON237807 | 2 | ON237895 | 11 |
| BLTV14 | TV | 22/05/2014 | 80 | A | 3,080 | B’ | 7 | III | ON237808 | 2 | ON237896 | 8 |
| BLTV18 | TV | 22/05/2014 | 80 | A | 3,080 | B’ | 7 | III | ON237809 | 2 | ON237897 | 8 |
| BLTV35 | TV | 22/05/2014 | 132.8 | A | 3,133 | B’ | 9 | I | ON237811 | 2 | ON237899 | 10 |
| BLTV42 | TV | 16/08/2014 | 700 | B | 3,700 | B’ | 3 | I | ON237812 | 5 | ON237900 | 31 |
| BLTV43 | TV | 16/08/2014 | 708 | B | 3,708 | B’ | 3 | I | ON237813 | 2 | ON237901 | 3 |
| BLTV44 | TV | 16/08/2014 | 714 | B | 3,714 | B’ | 12 | I | ON237814 | 2 | ON237902 | 13 |
| BLTV45 | TV | 16/08/2014 | 656 | B | 3,656 | B’ | 5 | I | ON237815 | 2 | ON237903 | 6 |
| BLTV46 | TV | 16/08/2014 | 544 | B | 3,544 | B’ | 10 | III | ON237816 | 2 | ON237904 | 11 |
| BLTV47 | TV | 16/08/2014 | 706 | B | 3,706 | B’ | 8 | I | ON237817 | 2 | ON237905 | 9 |
| BLTV50 | TV | 07/11/2014 | 485 | B | 3,485 | B’ | 2 | I | ON237819 | 2 | ON237907 | 2 |
| BLTV52 | TV | 07/11/2014 | 497 | B | 3,497 | B’ | 2 | I | ON237820 | 2 | ON237908 | 2 |
| BLTV54 | TV | 16/08/2014 | 707 | B | 3,707 | B’ | 28 | I | ON237821 | 2 | ON237909 | 32 |
| BLTV55 | TV | 16/08/2014 | 563 | B | 3,563 | B’ | 29 | II | ON237822 | 1 | ON237910 | 33 |
| BLTV56 | TV | 16/08/2014 | 531 | B | 3,531 | B’ | 29 | II | ON237823 | 1 | ON237911 | 33 |
| BLTV57 | TV | 16/08/2014 | 456 | B | 3,456 | B’ | 2 | I | ON237824 | 2 | ON237912 | 2 |
| BLTV58 | TV | 16/08/2014 | 658 | B | 3,658 | B’ | 30 | I | ON237826 | 2 | ON237914 | 34 |
| Pakse1 | Laos | 01/06/2017 | 4,000 | D | 6,000 | C’ | 26 | I | ON237833 | 2 | ON237921 | 29 |
| Pakse2, | Laos | 01/06/2017 | 4,400 | D | 6,400 | C’ | 26 | I | ON237834 | 6 | ON237922 | 37 |
| Pakse31, | Laos | 01/06/2017 | 4,000 | D | 6,000 | C’ | 15 | II |  | 1 |  | 16 |

Abbreviations: AG, An Giang; Bt, Ben Tre; CM, Ca Mau; ST, Soc Trang; TV, Tra Vinh.

Weight at catch was divided into four groups comprising A – Fingerling (<130 g), B – Sub-adult1 (365 – 714 g), C – Sub-adult2 (1,082 – 2,800 g) and D – Broodfish (3,100 – 9,000 g). Size-adjusted (by year at catch) was divided into three groups comprising A’ (2,000 – 3,000 g), B’ (3,000 – 4,000 g) and C’ (>5,000 g).

**Appendix S2** Haplogroups analyses

**Figure S1** The number of D-loop sequences in three haplogroups of different fish sizes (at sampling)

(Fingerlings: <130 g; Sub-adult1: 365 – 714 g; Sub-adult2: 1,082 – 2,800 g; Broodfish: 3,100 – 9,000 g)

**Figure S2** The number of D-loop sequences in three haplogroups of fish sampled in three years

**Figure S3** The number of D-loop sequences in three haplogroups of fish sizes adjusted by yeat at catch

(The fish weights at the sampling were adjusted to the estimated weight in 2019, the last year of sampling in this study. Because there is no information on age-length or age-weight relationships for *P. krempfi*, we estimated the weight of fish based on information from farmers who have cultured this species using wild-caught fingerlings (one year: 1 kg, 2 years: 2 kg, and 3 years: 3 kg)).
